# Robot‐Assisted Radical Prostatectomy in a Patient With Zinner Syndrome: A Case Report

**DOI:** 10.1002/iju5.70248

**Published:** 2026-09-07

**Authors:** Junji Yatsuda, Takashi Watanabe, Narumi Harada, Hidekazu Nishizawa, Toshiki Anami, Ryoma Kurahashi, Takanobu Motoshima, Masaya Baba, Yoji Murakami, Tomomi Kamba

**Affiliations:** ^1^ Department of Urology, Graduate School of Medical Sciences Kumamoto University Kumamoto Japan

**Keywords:** prostate cancer, robot‐assisted laparoscopic radical prostatectomy, Zinner syndrome

## Abstract

**Introduction:**

Zinner syndrome is a rare congenital urogenital anomaly characterized by seminal vesicle cysts, ejaculatory duct obstruction, and ipsilateral renal agenesis. Surgical treatment for prostate cancer in this setting has been reported in only 2 previous cases.

**Case Presentation:**

A 67‐year‐old man with congenital left renal agenesis presented with voiding dysfunction. Magnetic resonance imaging identified a 6‐cm seminal vesicle cyst and a Prostate Imaging‐Reporting and Data System category 4 prostatic lesion. Serum prostate‐specific antigen was 1.3 ng/mL. Prostate biopsy revealed Gleason score 3 + 3 = 6 adenocarcinoma. Robot‐assisted laparoscopic radical prostatectomy was performed using the posterior approach. Despite anatomical complexity and adhesions, the procedure was completed safely. Postoperative prostate‐specific antigen became undetectable, and the patient achieved pad‐free continence at 6 months.

**Conclusion:**

Robot‐assisted laparoscopic radical prostatectomy was safely performed for prostate cancer arising in Zinner syndrome. Robot‐assisted surgery is particularly valuable for complex anatomy caused by congenital anomalies.

## Keynote Message

This report demonstrates that Robot‐assisted radical prostatectomy can be performed safely and effectively in patients with prostate cancer complicated by Zinner syndrome despite substantial anatomical challenges. In this case, the robotic approach allowed precise dissection in a narrow and complex operative field, resulting in excellent perioperative, oncological, and functional outcomes for the patient. Clinically, this case provides useful guidance for surgeons managing rare congenital anomalies. From a broader perspective, such experience may contribute to improving surgical safety and postoperative quality of life in patients with uncommon but anatomically complex urologic diseases.

**Abbreviations**
CT: computed tomography
MRI: magnetic resonance imaging
PSA: prostate‐specific antigen
RARP: robot‐assisted laparoscopic radical prostatectomy

## 1 Introduction

Zinner syndrome is a rare congenital anomaly of the urogenital system, characterized by a classic triad of seminal vesicle cysts, ejaculatory duct obstruction, and ipsilateral renal agenesis [1, 2]. Although several reports have described surgical interventions aimed at alleviating symptoms associated with Zinner syndrome, surgical treatment for prostate cancer in patients with this condition remains uncommon. To the best of our knowledge, only two such cases have been reported in the literature [3, 4]. Herein, we report a case of robot‐assisted laparoscopic radical prostatectomy (RARP) for prostate cancer in a patient with Zinner syndrome. To our knowledge, this represents the third reported case of surgical treatment for prostate cancer in the setting of Zinner syndrome and the second to employ a robot‐assisted approach.

## 2 Case Presentation

**FIGURE 1:**
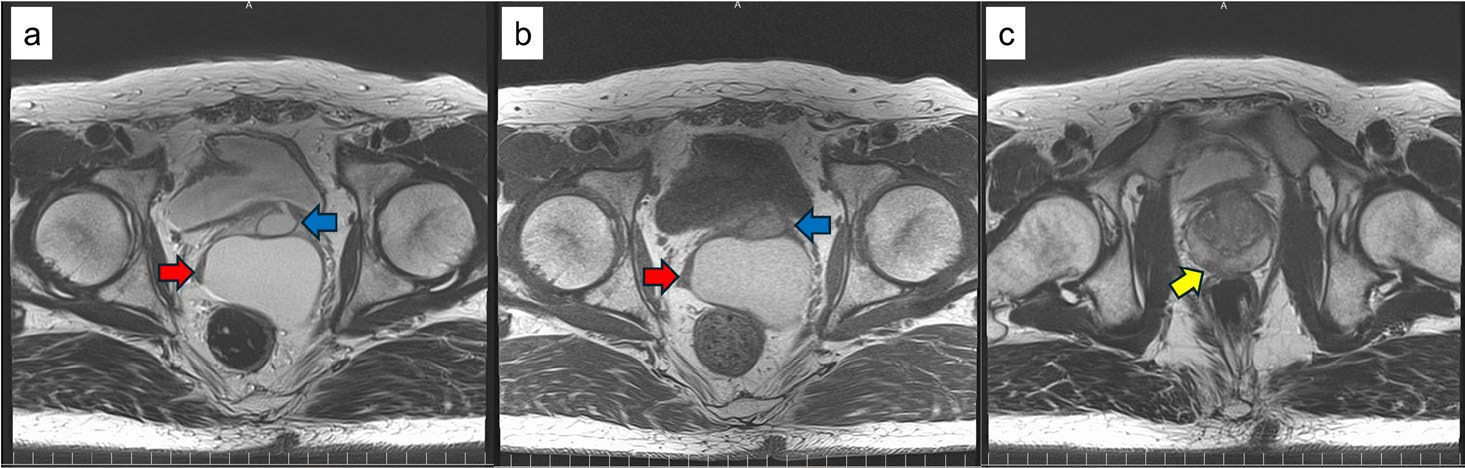
Red thick arrow indicates the suspected seminal vesicle cyst. Blue thick arrow, structure suspected to represent the ejaculatory duct. (a) MRI T2‐weighted image. The mass suspected to be a seminal vesicle cyst measured approximately 6 cm in diameter and demonstrated high signal intensity. It was continuous with a structure suspected to represent the ejaculatory duct. (b) MRI T1‐weighted image. The mass suspected to be a seminal vesicle cyst demonstrated relatively high signal intensity, suggesting that the internal fluid was viscous, such as mucin, or hemorrhagic. (c) MRI T2‐weighted image. A low‐signal‐intensity lesion was identified in the peripheral zone of the right lobe of the prostate, raising suspicion of malignancy (yellow thick arrow).

The patient was a 67‐year‐old man with an incidentally identified congenitally absent left kidney. He initially presented to a referring physician with complaints of voiding dysfunction, with a chief complaint of a sensation of difficulty urinating, and IPSS was 14, and the QoL score was 5. Ultrasonography revealed a mass lesion posterior to the bladder, prompting further evaluation with Magnetic Resonance Imaging (MRI). MRI revealed a 6‐cm lesion exhibiting homogeneously high signal intensity on T2‐weighted fat‐suppressed imaging (Figure 1a), which was contiguous with a structure adjacent to the ejaculatory duct, raising suspicion for a seminal vesicle cyst. The relatively high signal intensity on T1‐weighted imaging (Figure 1b) suggests that the cyst contents consisted of viscous fluid, such as mucin or hemorrhagic material. Concurrently, a lesion in the peripheral zone of the prostate was identified and classified as a Prostate Imaging‐Reporting and Data System category 4. The patient was referred to our department for further evaluation and management of both the posterior cystic lesion and suspected prostate cancer. Serum PSA was 1.3 ng/mL, and digital rectal examination revealed no palpable induration. Based on these findings, a diagnosis of Zinner syndrome with suspected prostate cancer was established, and the patient was scheduled for a prostate biopsy and left retrograde pyelography. Cystoscopy revealed a bulging lesion at the expected location of the left ureteral orifice; however, the orifice itself could not be identified, and retrograde pyelography was not performed. Prostate biopsy yielded a Gleason score of 3 + 3 = 6. Staging contrast‐enhanced Computed Tomography (CT) and bone scintigraphy revealed no evidence of distant metastasis. After a thorough discussion with the patient regarding treatment options, including active surveillance, RARP was selected, in part because voiding dysfunction was his primary complaint.

**FIGURE 2:**
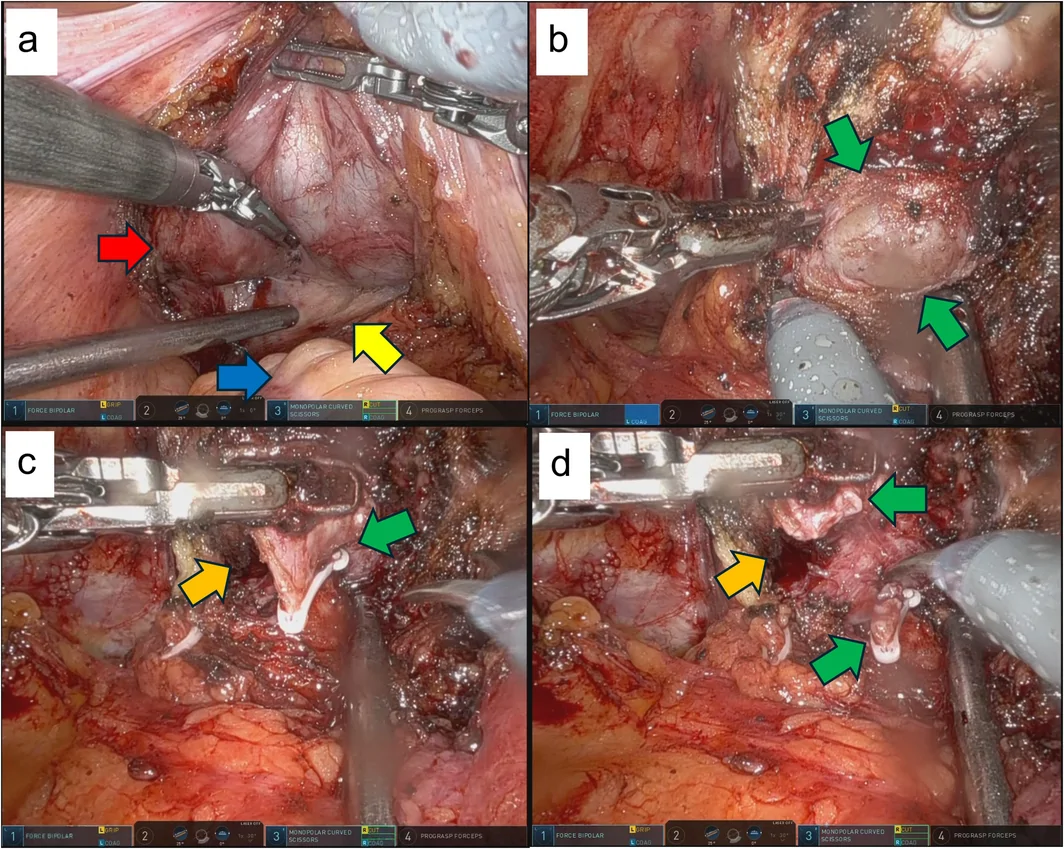
Red thick arrow, enlarged seminal vesicle. Blue thick arrow, sigmoid colon. Yellow thick arrow, the incised peritoneum. Green thick arrows, A vascular structure suspected to be a remnant ureter with ectopic orifice. Orange thick arrows, space created by the posterior approach. (a) A posterior approach was used to expose the enlarged seminal vesicle from its dorsal aspect. (b) A vascular structure continuous from the intramural bladder wall to the prostate was identified. (c) The vascular structure was clipped. (d) The clipped vascular structure was then transected.

**FIGURE 3:**
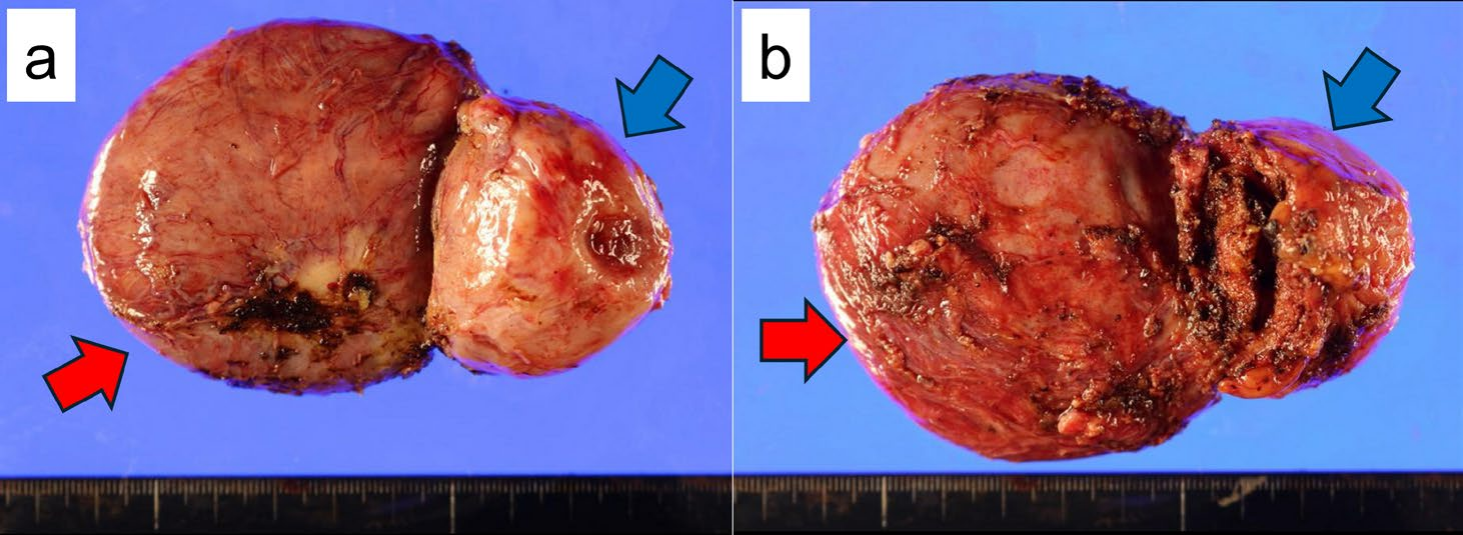
Red thick arrow indicates the resected seminal vesicle cyst. Blue thick arrow indicates the prostate. (a) Dorsal view; (b) ventral view.

RARP was performed using the posterior approach. The peritoneum was incised near the peritoneal reflection in the rectovesical pouch, and the cystic lesion was identified (Figure 2a). The posterolateral aspect of the cystic lesion was dissected toward the posterior surface of the prostate, followed by dissection between the cystic lesion and bladder. Because further posterior dissection was difficult and to avoid injury to the normal right ureter, the approach was converted to an anterior approach. After division of the bladder neck, the previously developed space was accessed, and a vascular structure extending from within the bladder wall to the prostate was identified (Figure 2b); it was clipped and divided (Figure 2c,d). The previously transected tubular structure corresponded to the enlarged ampulla of the vas deferens presumed preoperatively (Figure 1a). Given its course from the bladder wall to the prostate, it may have represented a remnant left ureter with an ectopic orifice. The remainder of the procedure was performed in a standard manner with bilateral nerve sparing. The total operative time was 4 h 59 min, console time was 3 h 53 min, estimated blood loss was minimal, and the specimen weight was 169 g (Figure 3). Pathological examination of the resected cyst revealed that it was lined with seminal vesicle epithelium, consistent with cystic transformation of the left seminal vesicle. The right seminal vesicle was normal in both size and histopathological findings. Regarding prostate cancer, a minute focus of Gleason score 3 + 3 = 6 adenocarcinoma was identified, consistent with the preoperative biopsy findings. PSA became undetectable immediately after surgery, voiding dysfunction resolved, and the patient achieved pad‐free continence at 6 months postoperatively. The preoperative IIEF‐5 score was 4, and the patient had no sexual activity before surgery. There was no change in eGFR between the preoperative and postoperative periods (55 and 54 mL/min/1.73 m^2^, respectively).

## 3 Discussion

Zinner syndrome is a rare congenital anomaly of the urogenital system, characterized by a classic triad of seminal vesicle cysts, ejaculatory duct obstruction, and ipsilateral renal agenesis (67%–75%) or renal hypoplasia. Fewer than 200 cases have been reported in the literature to date. The syndrome is typically asymptomatic and is often diagnosed incidentally. When symptomatic, clinical manifestations may include dysuria, perineal or lower abdominal pain (particularly when the cyst is large), hematospermia, epididymitis, and infertility. When such cysts are large, they may cause lower abdominal pain and may be palpable on rectal examination [1, 2]. In the present case, the patient presented with dysuria. The association between Zinner syndrome and malignant diseases, including prostate cancer, is considered rare.

Although several reports have described laparoscopic surgery [5, 6, 7], including robot‐assisted approaches [8, 9, 10, 11, 12], for Zinner syndrome, we identified only two cases in which radical prostatectomy was performed. One was performed via conventional laparoscopy [3], and the other employed a robot‐assisted approach, similar to our case [4]. In both cases, the atrophic ipsilateral kidney was resected simultaneously; however, in our case, resection was deemed unnecessary because the kidney was not visualized on CT. In both cases, seminal vesicle enlargement was mild, and neither involved a giant seminal vesicle cyst that would interfere with the operative field, as in the present case. In the present case, a marked enlargement of the seminal vesicle was observed. Given the limited operative field inherent to the posterior approach, combined with adhesions presumably attributable to chronic inflammation, the robot‐assisted surgical system—with its enhanced degrees of freedom of instrument articulation—was highly advantageous.

Based on our operative experience, we conclude that robot‐assisted surgery, which affords a magnified operative field and precise instrument control, is particularly valuable in cases where congenital anomalies render the anatomy complex or where dense adhesions are present.

## 4 Conclusion

RARP was performed for prostate cancer arising in the setting of Zinner syndrome. Although the case presented considerable anatomical challenges, the procedure was safely completed using a robot‐assisted surgical system.

## Funding

This work was supported by JSPS KAKENHI Grant Number 24K12442.

## Ethics Statement

Approved by the Institutional Review Board of Kumamoto University Hospital (Approval no. 1904). Informed consent was obtained from all participants and their guardians. Paperpal (Cactus Communications, Mumbai, India) was used to assist with language editing and manuscript preparation.

## Conflicts of Interest

The authors declare no conflicts of interest.

## Data Availability

The data that support the findings of this study are available from the corresponding author upon reasonable request.

## References

[1] A. Djidda , F. E. Badi , M. Sabiri , S. Elmanjra , S. Lezar , and F. Essodegui , “Zinner Syndrome,” European Journal of Case Reports in Internal Medicine 8 (2021): e002628. 10.12890/2021_002721 PMC8592662 34790618

[2] O. Adorisio , C. Orazi , L. M. Gregori , F. De Peppo , and M. Silveri , “Zinner Syndrome in Pediatric Patients: Rare Disease Leading to Challenging Management,” Frontiers in Pediatrics 12 (2024): 1353960. 38328345 10.3389/fped.2024.1353960 PMC10848245

[3] T. Spinos , I. Leotsakos , I. Katafigiotis , F. Nikitakis , and M. Karavitakis , “Laparoscopic Radical Prostatectomy Performed in a Patient With Zinner's Syndrome: The First Case Described in the Literature,” Cureus 15 (2023): e33764. 36793828 10.7759/cureus.33764 PMC9924158

[4] W. Narożański , M. Glembin , and D. Romanowicz , “Robot‐Assisted Radical Prostatectomy in a Patient With Zinner Syndrome,” European Journal of Case Reports in Internal Medicine 1 (2025): 110895. 10.1016/j.ijscr.2025.110895 PMC11848090 39919493

[5] T. Maehana , F. Fukuta , K. Kobayashi , M. Hirobe , T. Tanaka , and N. Masumori , “Laparoscopic Surgery for Seminal Vesicle Cysts and Ureterocele With Urination Disorder: A Case Report of Zinner Syndrome,” Journal of Endourology Case Reports 4 (2018): 35–38. 29588919 10.1089/cren.2018.0008 PMC5865617

[6] E. Corongiu , P. Grande , V. Olivieri , G. Pagliarella , and F. Forte , “Minimally Invasive Management of a Symptomatic Case of Zinner's Syndrome: Laparoscopic Seminal Vesiculectomy and Ipsilateral Nephroureterectomy,” Archivio Italiano di Urologia, Andrologia 91 (2019): 58–59. 10.4081/aiua.2019.1.58 30932434

[7] N. P. Kelly , A. Fuentes‐Bonachera , W. P. Shields , I. M. Cullen , and P. J. Daly , “Long‐Term Surveillance and Laparoscopic Management of Zinner Syndrome,” Case Reports in Urology 2021 (2021): 6626511. 33763284 10.1155/2021/6626511 PMC7964102

[8] S. Razdan , O. N. Kryvenko , and S. Razdan , “Robotic‐Assisted Laparoscopic Vesiculectomy in a Patient With Atypical Zinner Syndrome Presenting With Large Cyst Involving Bilateral Seminal Vesicles and Vasa Deferentia,” Urology Case Reports 18 (2018): 79–81. 29785379 10.1016/j.eucr.2018.03.014 PMC5958925

[9] M. C. Kiremit , O. Acar , A. A. Sag , et al., “Minimally Invasive Management of Zinner's Syndrome With Same‐Session Robot‐Assisted Seminal Vesiculectomy and Ipsilateral Nephroureterectomy Using a Single Geometry of Trocars,” Journal of Endourology Case Reports 4 (2018): 186–189. 30410997 10.1089/cren.2018.0066 PMC6222210

[10] L. Demaeyer , S. Holz , D. Pamart , S. Taylor , and M. Naudin , “Robotic Management of Painful Zinner Syndrome, Case Report and Review of Literature,” International Journal of Surgery Case Reports 73 (2020): 61–64. 32634620 10.1016/j.ijscr.2020.06.078 PMC7338681

[11] M. Cooper and L. Wiegand , “An Unusual Variant of Zinner Syndrome With Ureteral Ectopia From an Atrophied Supernumerary Kidney: A Case Report,” Urol. Case Rep. [Internet] 31 (2020): 101160. 32322509 10.1016/j.eucr.2020.101160 PMC7171451

[12] E. P. Kwenda , R. A. Locke , J. S. Archer , et al., “Robot‐Assisted Laparoscopic Resection of the Mesonephric Duct Remnant in a Patient With Zinner Syndrome,” Journal of Endourology Case Reports 6 (2020): 198–201. 33102726 10.1089/cren.2020.0020 PMC7580635

